# Numerical and Experimental Studies on the Explosive Welding of Tungsten Foil to Copper

**DOI:** 10.3390/ma10090984

**Published:** 2017-08-23

**Authors:** Qiang Zhou, Jianrui Feng, Pengwan Chen

**Affiliations:** State Key Laboratory of Explosion Science and Technology, Beijing Institute of Technology, Beijing 100081, China; zqpcgm@gmail.com (Q.Z.); 3120130059@bit.edu.cn (J.F.)

**Keywords:** W–Cu composite, explosive welding, numerical simulation, SPH method

## Abstract

This work verifies that the W foil could be successfully welded on Cu through conventional explosive welding, without any cracks. The microstructure was observed through scanning electron microscopy (SEM), optical microscopy and energy-dispersive X-ray spectrometry (EDS). The W/Cu interface exhibited a wavy morphology, and no intermetallic or transition layer was observed. The wavy interface formation, as well as the distributions of temperature, pressure and plastic strain at the interface were studied through numerical simulation with Smoothed Particle Hydrodynamics (SPH). The welding mechanism of W/Cu was analyzed according to the numerical results and experimental observation, which was in accordance with the indentation mechanism proposed by Bahrani.

## 1. Introduction

Due to the high sputtering resistance and low deuterium/tritium retention of tungsten as well as a high thermal conductivity, excellent welding properties and a relatively high strength of copper, the W–Cu bimetal constitutes a promising candidate for the plasma facing material (PFM) in fusion test reactors. These include the international thermonuclear experimental reactor (ITER) [1], where the W is utilized to resist the high heat load, the high flux low-energy ion and neutral particle irradiation along with Cu to transfer the heat loads to the water coolant. Consequently, the large difference in the coefficient of thermal expansion (CTE) and the elastic modulus between W and Cu creates significantly high stresses at the interface, decreasing the structure reliability under high heat loading. Various methods have been developed to join W and Cu, such as casting, e-beam welding, brazing, diffusion bonding (HIP), chemical vapor deposition (CVD) and selective laser melting (SLM) [2,3,4]. These methods have corresponding limitations such as lowering the allowable temperature of the interface, reducing the strength of copper substrate [5], low neutron resistance of the large-grained Cu and low cycle fatigue cracking [6].

Explosive welding is a solid-phase joining technique, in which several dissimilar metallic components can be welded with a strong metallurgical bond, whereas one of the components is driven by explosion to impact the others. The phenomenon of jet formation at the collision point is an essential condition for welding, which is generally accepted based on experimental evidence. The jet removes the oxide films and other contaminants from the mating surface, thereby making it possible for the atoms of the two materials to approach within inter-atomic distances under the high pressure (exceeding tens of GPa) induced by impact. The inter-atomic bonds are achieved with the effect of pressure and the deposited kinetic energy [7]. The high pressure generated instantaneously, as two plates colliding leads to severe plastic deformations of both materials. The kinetic energy of the impact plate is deposited at the interface in the form of plastic work; also, the partial plastic work is transformed into heat due to the adiabatic compression, leading to the melting of both materials at the impact surface. The heating rate during explosive welding is estimated to be of the order of 10^9^ K/s [8]. The high pressure far exceeds the yield strengths of both materials and makes the metals at the interface behave as a fluid. These metallic flows are instable and the wavy interface is formed during the interaction of flows, through which the two metals are “hooked up” with each other. Consequently, the bond of explosive welding is a combination of metallurgical bond and mechanical joint and is usually as strong or stronger than the weaker one of the components. This makes the explosive welding extremely suitable for the joining of metals with zero mutual solubility, such as W and Cu.

Due to its brittleness, W cannot withstand the large deformation during the acceleration process and the subsequent collision. Severe cracks occurred while the 1 mm thick W was explosively welded on the CuCrZr [9]. However, brittle behavior is not intrinsic to W, the soluble interstitial impurities segregating along the grain boundaries (GBs) render the GBs the weak link, leading to the grain boundary embrittlement [10]. The ductility may be improved if the average impurity concentration at the GBs can be reduced. This implies that a thin W plate may be more ductile since the impurity concentration is reduced along the rolling direction, due to the grain elongation. The thin W plate utilization may be an appropriate method to avoid cracking. Otherwise, coating a thin plate on the surface of the parent plate will produce certain special properties required in the industry, such as corrosion resistance, hardness, high temperature resistance, suitable frictional properties or special electrical properties. In contrast, the explosive welding of quite thin metallic foils is hard to achieve, due to the destruction under a high strain rate, requiring a proper selection of parameters.

The underwater explosive welding process was explored and has been successfully applied to clad metallic foils, including W foils. Mori et al. [11] cladded the 0.2 mm thick, 50 mm square W foils onto ferritic steel F82H plate through underwater explosive welding. Thus, a partial W foil with an area of 30 mm^2^ square was welded and micro-cracks were observed at the interlayer, due to the residual stress caused by the CTE mismatch. Manikandan et al. [12] cladded pure W foils on copper through underwater explosive welding, whereas occasionally cracks were observed in the experiments ranging from 0.1 mm to 0.5 mm of the tungsten foil. The authors claimed that the reflected tensile wave caused the plane crack in 0.1 mm W foil and the bending forces during acceleration caused the grain boundary cracking in 0.5 mm thick W. For underwater explosive welding, however, it was difficult to isolate the explosive from the water and the use of inclined set-up had corresponding limitations in the welding of large-sized plates [13].

The purpose of this paper was to fabricate a crack free W–Cu bimetal with W foils through explosive welding in the open air. Furthermore, the morphology of the interfacial microstructures of the W/Cu joint was investigated. A numerical simulation was also conducted to offer insights into the wavy interface and vortex formations.

## 2. Experimental Procedure

**Figure 1:**
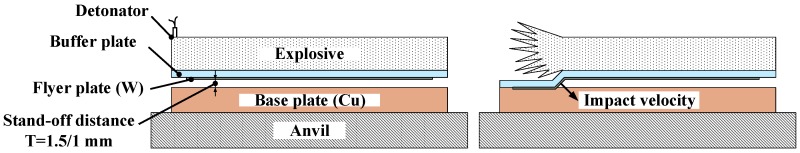
Schematic illustration of explosive welding of thin W and Cu plates.

A pure W plate was utilized as the flyer plate and pure Cu plates were used as the base plate. The size of the clad plate was 100 mm × 50 mm. The thickness of the Cu plates was 10 mm and the thickness of the W plate varied from 0.1 mm to 0.5 mm. Also, 2024 Al/pure Cu sheets of 2 mm in thickness were utilized as buffer layers between the explosive and the W plate, as presented in Figure 1. The compacted sand was utilized as an anvil to avoid the elastic recoil. A powder explosive named expanded ammonium nitrate (EAN) of 10 mm in constant thickness and of 1.0 g/cm^3^ in packing density was employed in all experiments. The EAN explosive was a mixture of puffed ammonium nitrate, diesel and saw powders; perlite powders are also added to the EAN as inert materials to lower the detonation velocity. As a non-ideal explosive, the EAN detonation velocity was dramatically affected by the packing density and thickness. In this study, the average detonation velocity with a fixed thickness of 10 mm was measured to be 2600 m/s by the resistance change measurement of a cable probe with the Handi Trap^II^ VOD recorder (MREL Group of Companies Limited, Kingston, ON, Canada). The cable probe was embedded in the explosive and was shortened by the plasma during detonation.

Generally, the preset stand-off distance should be twice the flyer thickness, which would be sufficiently high for the flyer to be accelerated to the maximum velocity [14]. In this case, the stand-off distance should be at least 4 mm, taking account of the buffer plate thickness. However, the actual stand-off distance was below 1.5 mm, in order to lower the impact velocity and bending angle, avoiding the fracture of W from large deformation during the acceleration. The dominating parameters of explosive welding were the impact velocity *V_P_* and the collision angle *β*; a relationship existed between these parameters, which is given by [15]:

(1) $$V_{P}=2D\sin\frac{\beta }{2},$$

where *D* is the detonation velocity. It meant that the only parameter left unknown on the equation is the collision angle once the detonation velocity *D* is measured and the impact velocity *V_P_* is determined using numerical simulation. In this work, the impact velocity was altered by the stand-off distance and the E/M value adjustments, whereas the E/M value is the mass ratio of explosive to flyer and buffer plate, presenting the explosion energy per unit mass. The impact velocity was determined by the simulation presented in the following section. The dynamic parameters of the welding are listed in Table 1. The interfacial microstructures were characterized through scanning electron microscopy (SEM, JEOL Ltd., Tokyo, Japan) and light microscopy (ZEISS, Oberkochen, Germany).

## 3. Weldability Window Calculations

**Figure 2:**
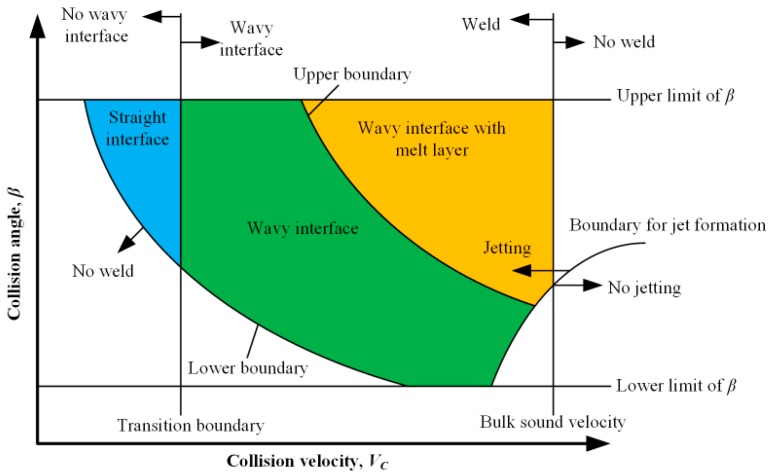
Weldability window concept for explosive welding.

The weldability window concept has been introduced to predict whether or not bonding will occur under different welding conditions. Wittman and Carpenter [16] and Deribas [17] developed an applicable weldability window in which the collision angle *β* was plotted against the collision velocity *V_C_*, as shown in Figure 2. There also exists the relationship between *V_C_* and the impact velocity *V_P_* in the parallel geometry, which is given by [15]:

(2) $$V_{C}=\frac{V_{P}}{2\sin\frac{\beta }{2}}$$

where *β* is the collision angle.

The right boundary defines the conditions to the formation of a jet at the collision point. Walsh stated that the *V_C_* should be smaller than the sound speed of the materials to be welded. However, Abrahamsen [18] suggested that *V_C_* is a weak function of the collision angle *β*, as follows:

(3) $$V_{C}=\frac{\beta }{10}+5.5$$

The lower boundary relates to the achieved impact pressure at the collision point exceeding the yield stress of the materials, so that plastic deformation occurs. Deribas [17] developed an equation for this limit:

(4) $$\sin\beta =k\sqrt{\frac{H_{V}}{\rho V_{c}^{2}}}$$

where *H_V_* is Vickers hardness of flyer, *ρ* is material density and are as mentioned above. Constant *k* takes values between 0.6 for clean surfaces, and 1.2 for imperfectly cleaned surfaces.

The left boundary is also called transition boundary, related to the formation of a wavy interface. Kuzmin and Lysak [19] experimentally proved that this boundary depends not only on *V_C_*, but also on the collision angle *β*. Therefore, Crossland and Williams [20] and Wittman [21] proposed a dynamic plasticity criterion for transition from straight to wavy interface, as follows:

(5) $$\tan\beta =1.14\sqrt{\frac{H_{V}}{\rho V_{c}^{2}}}$$

where the parameters are the same as before.

The upper boundary is considered as maximum impact velocity which avoids the formation of an interfacial melted layer. Deribas [17] calculated the upper boundary as:

(6) $$\sin\frac{\beta }{2}=\frac{1}{2h^{0.25}V_{C}^{1.25}}\sqrt{\frac{E}{3\rho \left(1-2\vartheta \right)}}$$

where *E* and *ν* are the elastic modulus and Poisson’s ratio, and *h* is the thickness of the flyer. Other parameters are the same. In addition to the mentioned boundaries, Bahrani and Crossland [22] experimentally obtained a lower limit of 2–3° and an upper limit of 31° for collision angle *β* in parallel geometry; below and above these angles, welding is impossible. Material properties used for weldability window calculation are presented in Table 2.

## 4. Numerical Simulation

### 4.1. Smoothed Particles Hydrodynamics (SPH)

The simulation of explosive welding was carried out using the smoothed particles hydrodynamics (SPH) method with the Ansys LS-DYNA software (Ansys 14.5, LSTC, Livermore, CA, USA). SPH is one method of gridless Lagrangian hydrodynamics with particle utilization and is applied to solve problems with large deformations and moving discontinuities [23], such as in high velocity impact welding [24] and metal jets formed by shaped charges [25]. In the SPH method, a collection of particles was utilized to represent a given body. The governing equations of derivatives of the conservation laws were discretized by an integral form given as:

(7) $$f\left(x\right)=\int_{-2h}^{2h}f\left(x^{\prime }\right)W\left(x-x^{\prime },h\right)dx^{\prime }$$

where vectors *x* and *x*′ are the spatial locations of the particle of interest and its neighboring particles within the smooth length *h* from it respectively. Also, the *h* determines the number of particles that affect the interpolation for a particular point. In this method, close particles contribute significantly compared to distant particles. The Kernel Function *W* is utilized to integrate the derivatives, which is analogous to the shape function in the finite element method, whereas it does not require connectivity between particles. Therefore, the particles are able to move relative to each other in the domain of the simulation, thus allowing the jetting and wavy interface during explosive welding to be numerically predicted.

### 4.2. Modelling

**Figure 3:**
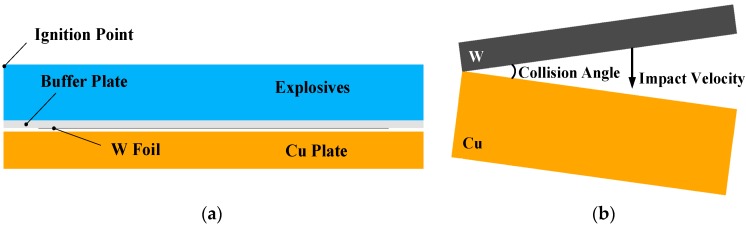
Schematic of Smoothed Particle Hydrodynamics (SPH) models: (**a**) the first stage; (**b**) the second stage.

In this paper, the simulation was divided into two stages due to the small thickness of the tungsten foil (<0.5 mm) in comparison to the dimensions of the other elements. At the first stage, the acceleration of the W foil by detonation of the explosive was simulated. The impact velocity and collision angle were measured and used at the next stage of simulation. The numerical models were in the plane formulation with the dimensions equal to the experimental conditions, as shown in Figure 3a. The particle size was 0.1 mm, which allowed the problem to be solved at acceptable spatial resolution and corresponding computation duration.

At the second stage, the collision of W and Cu plate in the explosive welding process was simulated using an oblique impact configuration model by the SPH method, as shown in Figure 3b. The direction of impact velocity was assumed to be perpendicular to the bisector of collision angle, which was widely accepted in parameter calculation [15]. The impact velocity was utilized as the initial particle velocity of the W foil and the collision angle was set to be the initial angle between W foil and Cu plate. The particle size was set as 1 μm and a total of 250,000 particles were utilized. The purpose of the simulation was to investigate the mechanism of wavy interface formation and the distributions of temperature and pressure.

The free boundary was set to both models in accordance with the physical conditions, allowing the stress wave to be reflected from the free surface.

At the first stage of simulation, the Chapman–Jouguet (C-J) detonation process of the explosive was modeled through the material model “MAT_HIGH_EXPLOSIVE_BURN”. The expansion of the detonation products was represented through the Jones–Wilkins–Lee equation of state (JWL EOS). The JWL coefficients and the main C-J characteristic values are given in Table 3. The JWL EOS is as follows:

(8) $$P=A\left(1-\frac{\omega }{VR_{1}}\right)exp\left(-R_{1}V\right)+B\left(1-\frac{\omega }{VR_{2}}\right)exp\left(-R_{2}V\right)+\frac{\omega E}{V}$$

where, *A*, *B*, *R*_1_ and *R*_2_ are constants. *P* is the pressure, *E* is the specific internal energy, and *V* is the relative volume. The *ω* is the Grüneisen parameter. Due to the lack of experimental data, the JWL coefficients of EAN were obtained by fitting the *P–V* curve of detonation products. The *P–V* curve was calculated through the following equation, which is deduced by neglecting the cold part of the EOS equation proposed by Baum [26]:

(9) $$P={\left(\frac{k}{k+1}\frac{1}{\rho_{0}}\right)}^{k}\frac{\rho_{0}^{k\;+1}D^{2}}{k+1}\frac{1}{V^{k}}$$

where *D* is the detonation velocity, *ρ*_0_ is the density of the explosive, *V* is the relative volume and *k* is the adiabatic exponent of the detonation products. The adiabatic exponent *k* is calculated through the Kamlet equation [27] and takes a value of 2.46.

The material behavior was modelled using a Johnson–Cook constitutive relationship:

(10) $$\sigma =\left(A+B\varepsilon_{eff}^{n}\right)\left(1+C\ln\dot{\varepsilon }\right)\left(1-T^{*m}\right)$$

where *σ* is the flow stress, $\varepsilon_{eff}$ is the effective plastic strain, $\dot{\varepsilon }={\dot{\varepsilon }}_{eff}/{\dot{\varepsilon }}_{0}$ is the plastic strain rate, $T^{*}=\left(T-T_{room}\right)/\left(T_{melt}-T_{room}\right)$ is the homologous temperature, where *T_room_* is the room temperature and *T_melt_* is the melting point. *A*, *B*, *C*, *m* and *n* are the material constants. The pressure of the material was obtained through the Grüneisen EOS. It is given by

(11) $$P=\{\begin{array}{c} \frac{\rho_{0}C_{0}^{2}\mu \left[1+\left(1-\gamma_{0}/2\right)\mu \right]}{{\left[1-\left(S-1\right)\mu \right]}^{2}}+\gamma_{0}E;\;\mu \leq 0\\ \rho_{0}C_{0}^{2}\mu \mu +\gamma_{0}E;\;\mu >0 \end{array}$$

where *C*_0_ is the intercept of the Hugoniot curve, *S* is the coefficient of the slope of the Hugoniot curve, $\gamma_{0}$ is the Grüneisen gamma, $\mu =\rho /\left(\rho_{0}-1\right)$, ρ is the density, $\rho_{0}$ is the initial density and *E* is the specific internal energy. The Johnson–Cook parameters for W [28] and Cu [29], as well as the Grüneisen parameters for W and Cu [30], are given in Table 4.

## 5. Results and Discussion

**Figure 4:**
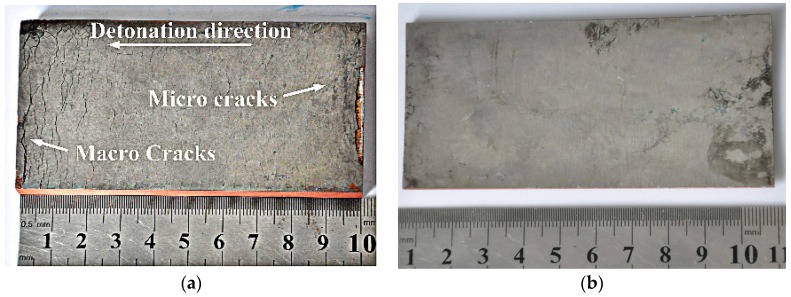
Photograph of recovered W–Cu plates: (**a**) 0.5 mm thick W (sample T-1); (**b**) 0.1 mm thick W (sample T-8).

The photographs of the welded plates recovered subsequent to explosive welding are shown in Figure 4. Figure 4a presents the apparent cracks and peeling at the edge of the 0.5 mm thick W. It was discovered that the annular micro-cracks near the ignition point evolved into macro-cracks at the other edge along the detonation direction. These cracks distributed near both ends, were transverse cracks induced by tensile wave, the formation of which will be discussed below. The micro-cracks near the ignition point displayed a concentric distribution, whereas the macro-cracks at the opposite edge distributed in parallel. This crack trend was consistent with the shape of the detonation wave, shown as concentric arcs near the ignition point and as parallel lines far away from the ignition point. For the 0.1 mm W foil, few cracks could be observed, indicating that the W foil could withstand large deformation and high strain rate due to its huge improvement in ductility.

**Figure 5:**
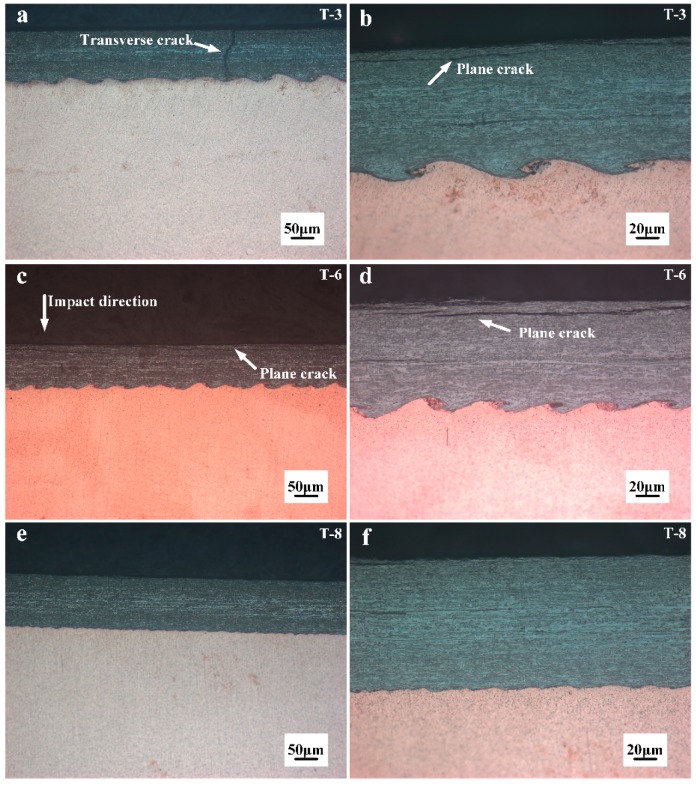
Comparison of interfacial morphologies among different samples: (**a**,**b**) T-3; (**c**,**d**) T-6; (**e**,**f**) T-8.

**Figure 6:**
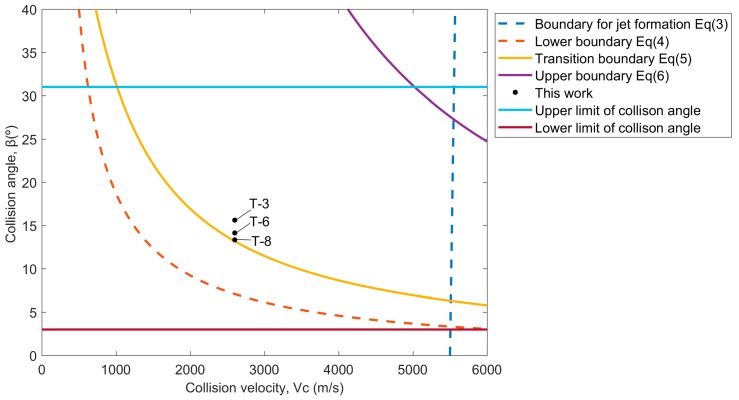
The calculated weldability window of 0.1 mm W–Cu for explosive welding.

Figure 5 shows the interface optical micrograph of the 0.1 mm W/Cu with various collision angles and impact velocities. The wavy structure shown in Figure 5b,d is a typical wavy interface of the explosively welded composites, which has been proved to be an indication of high bonding strength by numerous references, such as in copper/stainless steel [31], CuCrZr/316LN-IG [32], and stainless steel/carbon steel [33]. During explosive welding, the mechanical energy released during strong impact resulted in intense plastic deformation at the interface. The amplitude and wavelength of the wavy morphology could be used to estimate the degree of deformation as the plates collide. The transition from a wavy interface of T-3 to a straight one of T-8 was related to a decrease in the plastic strain and shear stress, as the impact velocity decreased from 706.6 m/s to 604.1 m/s. The calculated weldability window for 0.1 mm W/Cu and the experimental results are shown in Figure 6. The conditions of all the experiments are above the lower boundary, and T-8 with a straight interface is located on the transition boundary, above which we end up with wavy interfaces for T-3 and T-6. The prediction agrees well with the experimental results, and Equation (5) can reliably predict the transition from the straight to wavy interface.

**Figure 7:**
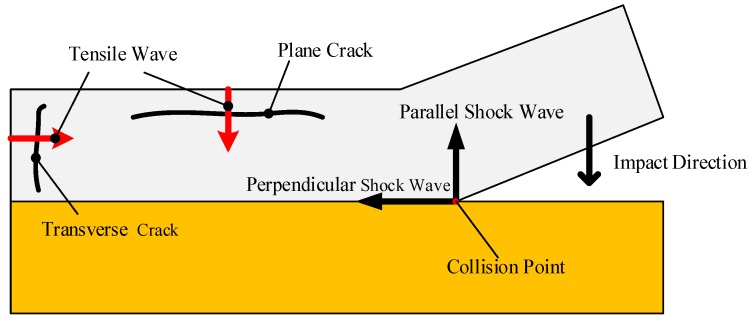
Schematic illustration of crack formation during collision.

Cracks were still observed for the samples cladded with higher impact velocity, as shown by Figure 5a,c. It was notable that the cracks displayed two morphologies: the plane crack and the transverse crack, which were caused by the tensile wave. As the buffer plate with the attached W foil impacted the Cu plate, the shock waves were formed at the interface instantaneously and propagated in the W plate along two directions: parallel to and perpendicular to the impact direction. When the perpendicular shock wave reached the free surface, a tensile wave was reflected, leading to transverse cracking. Similarly, the parallel shock wave reached the interface between the W and the buffer plate, where a tensile wave was reflected due to the impedance mismatch, leading to plane cracking. The schematic illustration of the formation of cracks is presented in Figure 7. The tensile wave intensity depended on the kinetic energy loss at the interface upon collision, which was deposited in the form of plastic work and shock wave. Through lowering the impact velocity from 706.6 m/s to 639 m/s, the tensile wave intensity was decreased and the transverse cracks were eliminated, as shown in Figure 5c. However, the W foils are quite susceptible for in-plane crack formation due to the layered stacking of elongated W-grains during rolling [34], and the plane cracks still existed in T-6. Consequently, the Cu plate was used as the buffer plate instead of Al for sample T-8, to lower the tensile wave intensity by reducing the impact velocity and the mismatch in impedance between the W foil and the buffer plate. As presented by Figure 5f, the plane cracks were finally eliminated. Moreover, the reduction in impact velocity also led to a straight interface. It was indicated that the crack-free W foil and the Cu could be successfully joined through the explosive welding technique. The harmful effect of the tensile wave could be eliminated by the buffer plate material adjustment. In addition, as shown in Figure 6, the sample T-6 with plane cracks is located slightly above the transition boundary. It means that the parameters for explosive welding of W–Cu should be adjusted precisely to achieve a wavy interface without cracks.

**Figure 8:**
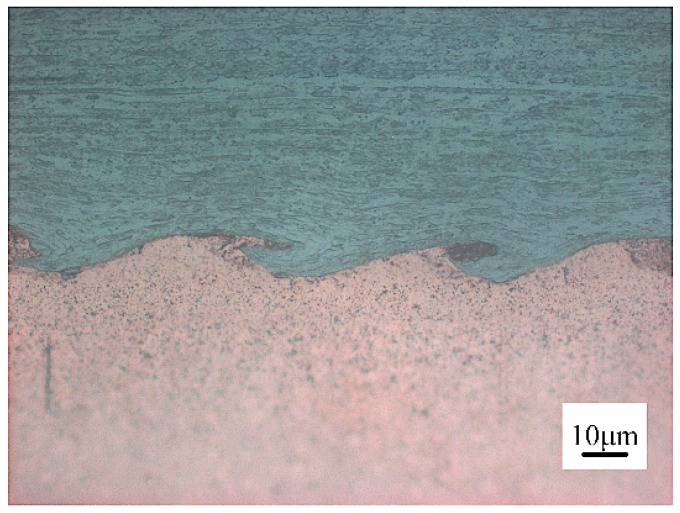
Optical microstructure of the etched W–Cu interface of T-6.

**Figure 9:**
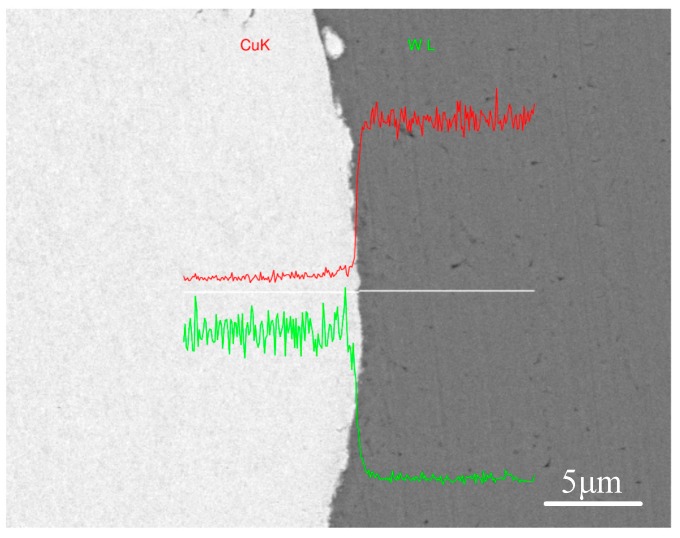
Chemical composition distribution across the W–Cu interface of T-6.

**Figure 10:**
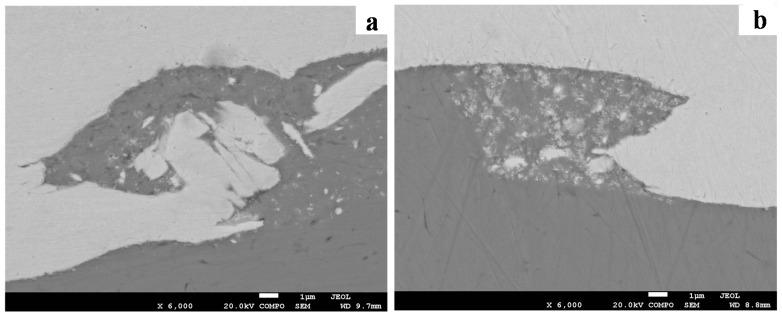

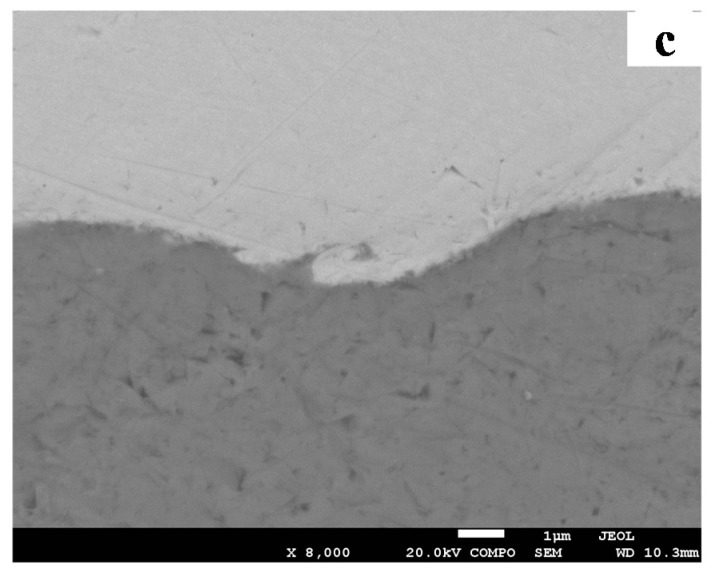
SEM images of the W–Cu interface: (**a**) T-3; (**b**) T-6; (**c**) T-8.

**Figure 11:**
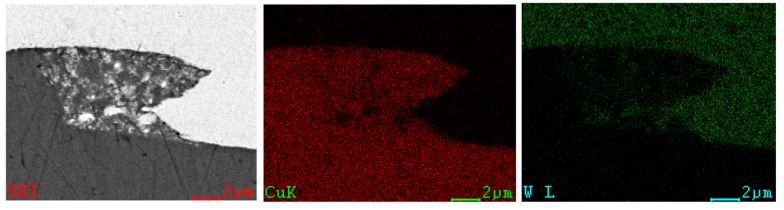
EDS-derived elemental maps for Cu and W of the W/Cu interface of T-6.

Figure 8 provides a close-up of the etched interface of W–Cu, demonstrating that the W grain afar from the interface retained the elongation parallel to the rolling direction, whereas the W grain adjacent to the interface was drawn into the vortex by the intruding Cu flow. The energy-dispersive X-ray spectrometry (EDS, Oxford Instruments, OAbingdon, UK) line scanning (plotted in Figure 9) exhibited a distinct interface of the W and Cu, where no intermediate layer was formed due to the zero-mutual solubility of W and Cu. The enlarged views of the interfaces of the W–Cu bimetal welded with different impact velocities are presented in Figure 10. Figure 10a shows the severe deformation and fracture of W under the Cu jet penetrating. The deformed W appeared to be stirred by the Cu jet and moved counter-clockwise to form a vortex. The process was verified by the following simulation. Actually, the W was crushed into tiny pieces by the Cu jet as the penetration started. Consequently, the W was deformed under the penetration and the collision, and stirred by the Cu jet with residual kinetic energy, forming the vortex shown in Figure 10a. With a lower impact velocity, the kinetic energy of the Cu jet was not sufficient to support a severe deformation of W, due to the dissipation during crushing. It explains the morphology shown in Figure 10b, where the vortex size was lower than the vortex size shown in Figure 10a and numerous fine W particles were embedded into the Cu matrix. The elemental mapping presented in Figure 11 verified that the fine particles were of pure W, not intermetallic. Such flocculent W pieces were also observed in the compaction of W–Cu powders under intense shock loading [35]. As the impact velocity decreased further, the deformation of W and the kinetic energy of the Cu jet were significantly weakened, resulting in the small hump shown in Figure 10c.

**Figure 12:**
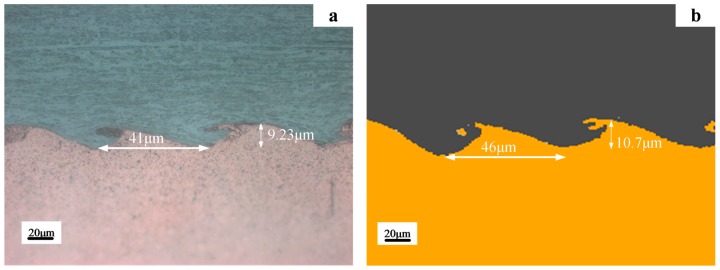
Comparison of experimental and numerical results for T-6 with impact velocity of 639.8 m/s: (**a**) experimental result; (**b**) numerical result.

Figure 12 presents the morphology comparisons between the experimental observation and the numerical simulations for the T-6 with the identical impact velocity and impact angle, of 639.8 m/s and 14.14°, respectively. The predicted wave morphology agreed with the experimental results, and the predicted amplitude whereas wave length was 10.7 μm and 46 μm while the experimental results were 9.23 μm and 41 μm, respectively. It was validated that the present modelling was feasible and could provide a useful insight into the plastic deformation and temperature distribution, which could not be observed or measured experimentally.

**Figure 13:**
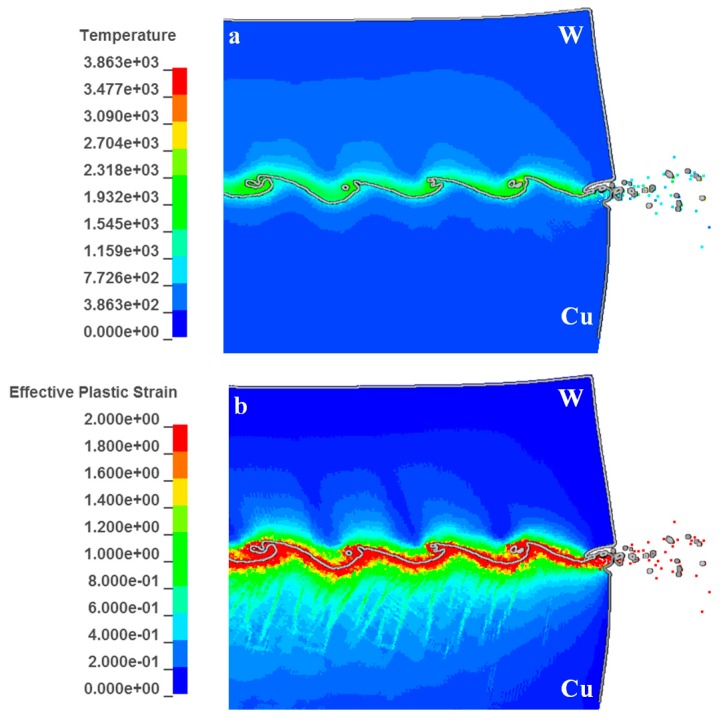
(**a**) Distributions of high temperature; (**b**) distribution of plastic strain at the interface.

Figure 13 presents the predicted high temperature and plastic strain distributed along the wave interface. The mechanical energy released during strong impact resulted in intense plastic deformation, friction and shear of the two materials, which was eventually converted into heat accumulation [36]. Since the process occurred over microseconds, there is no time for heat transfer away from the interface. The temperature was highly localized in the vortex and near the interface. Figure 13a exhibits that the high temperature region at the W side was in the range of 600–1700 K, with a thickness of ~7 μm, whereas the region at the Cu side was in the range of 600–1200 K, with a thickness of ~2 μm. A higher amount of energy was accumulated at the W side due to its lower conductivity compared to Cu, leading to lower energy loss by thermal conduction. The highest temperatures located in the vortex were 2400–3000 K for W and 1700–2100 K for Cu, indicating that the W did not melt during the formations of the jet and the vortex. In other words, all jets originated from the Cu side. This was verified by the experimental results, as shown in Figure 10a,b.

**Figure 14:**
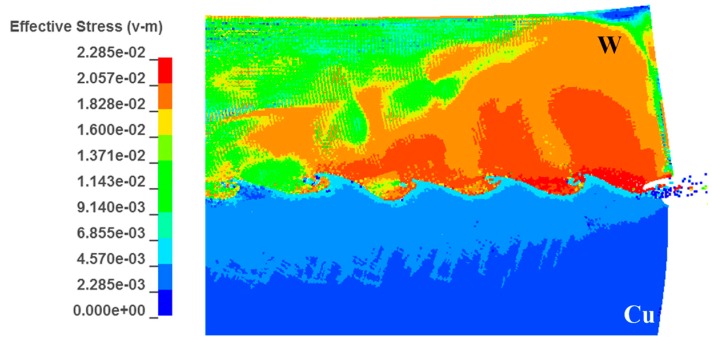
Distribution of equivalent stress at the W/Cu interface.

Figure 13b presents the plastic strain distribution at the interface. It shows that the distribution of high temperature is generally consistent with the high strain region, in accordance with the fact that heat is transformed from plastic work during the adiabatic compression. However, in this case, the region with higher temperature at the W side does not present higher strain. A similar trend was also observed by Zhang et al. [37]. Figure 14 presents the equivalent stress for the W adjacent to the interface at 2~2.2 GPa, 3~4 times the stress for the Cu side, 450~685 MPa. As shown in Figure 13b, the strain at the W side, which exceeded 0.8, was 0.4~0.5 times the strain for the Cu side, from which it could be deduced that the plastic work deposited at the W side was still higher than the Cu side. This explained why the region with higher temperature presented lower strain.

**Figure 15:**
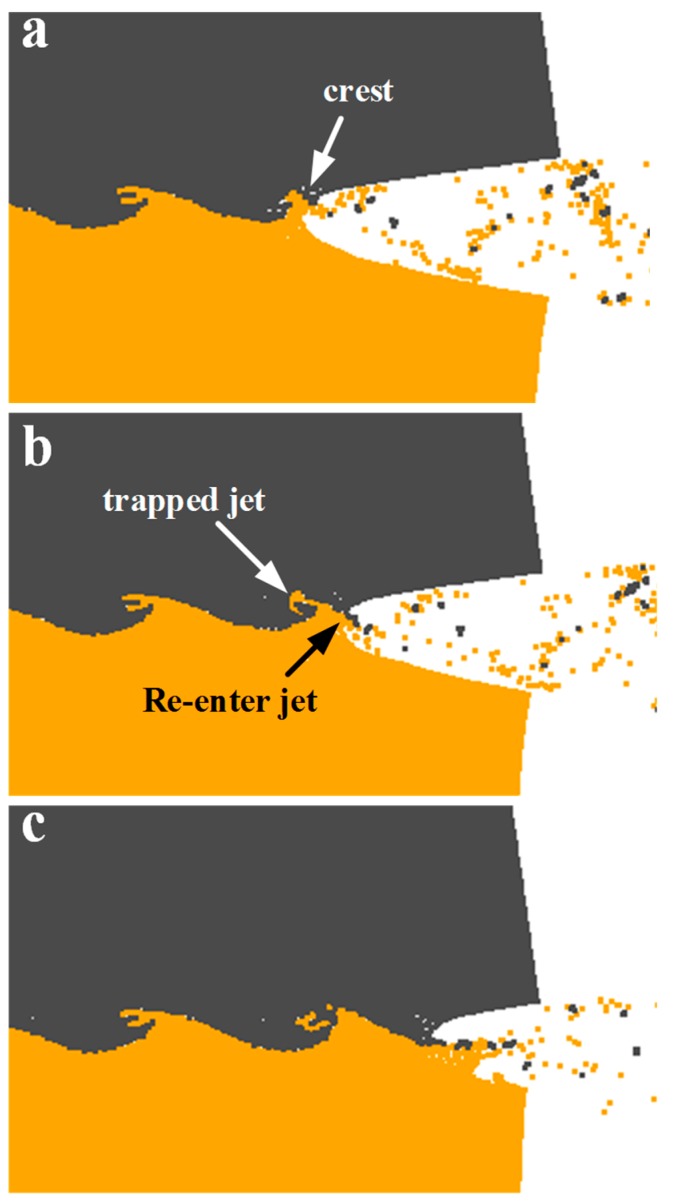
Formation of wave structure during explosive welding simulation: (**a**) the formation of crest; (**b**) the jet divided by the crest; (**c**) the newly formed jet.

Figure 15 presents the vortex formations in the simulation, which was in accordance with the indentation mechanism proposed by Bahrani [38]. The Bahrani mechanism has been questioned due to the assumption that the jets were all from the flyer plate, which was not in agreement with the facts. For the common metallic plates, the jet originates from both plates as the differences in density and melting point are not considerable. For the case of W and Cu, their physical properties differ dramatically, especially in the melting points. This implied that the jets might all originate from the Cu side during the collision by the W flyer, which makes the Bahrani mechanism reasonable. It is known [39] that if the colliding metals differ greatly in density, the discrete jet does not move along the bisector of the collision angle but is deflected towards the denser (in our case, W). The W plate deformed under the penetration by the Cu jet and consequently a crest was formed (Figure 15a). Subsequently, the Cu jet was divided into two parts by the crest: one part was the trapped jet behind the crest and the other part was the re-enter jet, as shown in Figure 15b. The trapped jet was completely choked, leading to a counter-clockwise movement and intense stirring with the severely deformed W. A backward vortex was then produced. Also, the re-enter jet was forced to move towards the Cu side by the crest, which was elongated as the trapped jet kept penetrating the W plates. Finally, the re-enter jet fell into the newly formed jet and produced another crest repeatedly, as shown in Figure 15c.

## 6. Conclusions

In this study, the crack-free W–Cu bimetal with W foils utilization was successfully fabricated through explosive welding in the open air. The cracks caused by the tensile wave could be eliminated by the impact velocity lowering and the mismatch reduction in impedance between the W foil and the buffer plate. The W–Cu interface transformed from a wavy morphology to a straight morphology as the impact velocity decreased. The numerical simulation through the SPH method captured the wavy morphology well, providing accurate results with respect to wavelength, amplitude, pressure and temperature at the interface. The simulated result agreed well with the experimental result, and both verified that the jets were all from the Cu side. The wavy interface and the vortex were formed with severe deformation and fracture of W, under the Cu jet penetration, which was in accordance with the indentation mechanism proposed by Bahrani.

## Figures and Tables

**Table 1:** Dynamic parameters of welding.

No.	W Foil Thickness (mm)	E/M	Stand-Off Distance (mm)	Cu Plate Thickness (mm)	Buffer Plate Thickness	Impact Velocity (m/s)	Collision Angle (°)	Status
T-1	0.5	0.67	1.5	10	2 mm Al	653.4	14.44	Cracks
T-2	0.2	1.08	1.5	10	2 mm Al	674.5	14.91	Cracks
T-3	0.1	1.37	1.5	10	2 mm Al	706.6	15.62	Few cracks
T-6	0.1	1.37	1.0	10	2 mm Al	639.8	14.14	Few cracks
T-8	0.1	0.50	1.0	10	2 mm Cu	604.1	13.34	No cracks

**Table 2:** Material properties used for weldability window calculation.

Material	Density (g/cm^3^)	Vicker Hardness (HV)	Elastic Modulus (GPa)	Poisson’s Ratio *ν*
W	19.23	560	410	0.28

**Table 3:** Jones–Wilkins–Lee (JWL) coefficients and C-J parameters of expanded ammonium nitrate (EAN).

Density (g/cm^3^)	Detonation Velocity (m/s)	C-J Pressure (GPa)	*A* (GPa)	*B* (GPa)	*R*_1_	*R*_2_	*ω*	*E* (GJ/m^3^)
1.0	2600	2	100	4.336	7.141	2	0.2	3

**Table 4:** Parameters of Johnson–Cook equation and Grüneisen EOS for W and Cu.

Material	Density (g/cm^3^)	*A* (MPa)	*B* (MPa)	*n*	*C*	*m*	*T_melt_* (K)	*C*_0_ (km/s)	*S*	Grüneisen Gamma
W	19.23	330.17	1027.4	0.018752	0.034454	0.40552	3683	4.06	1.20	1.78
Cu	8.94	90	292	0.31	0.025	1.09	1356	3.91	1.51	1.52
